# Management of the Hepatic Lymph Nodes during Resection of Liver Metastases from Colorectal Cancer: A Systematic Review

**DOI:** 10.1155/2008/684150

**Published:** 2007-09-04

**Authors:** Kurinchi S. Gurusamy, Charles Imber, Brian R. Davidson

**Affiliations:** University Department of Surgery, UCL and Royal Free Hospital NHS Trust, Royal Free and University College School of Medicine, London NW3 2QG, UK

## Abstract

*Background*. Hepatic lymph node involvement is generally considered a contraindication for liver resection performed for colorectal liver metastases. However, some advocate hepatic lymphadenectomy in the presence of macroscopic involvement and others routine lymphadenectomy. The aim of this review is to assess the role of lymphadenectomy in resection of liver metastases from colorectal cancer. *Methods*. Medline, Embase and Central databases were searched using a formal search strategy. Trials with survival data with a minimum follow-up of 1 year were considered for inclusion. Meta-analysis was performed using Revman. *Results*. A total of 4230 references were identified. Ten reports of nine studies including 926 patients qualified for the review. The prevalence of nodal metastases after routine lymphadenectomy was 16.3%. The overall 3-year and 5-year survival rates in node-positive patients were 9/151 (11.3%) and 2/137 (1.5%), respectively, compared to 3-year and 5-year survival rates of 424/787 (53.9%) and 246/767 (32.1%) in node-negative patients. The odds ratios for 3-year and 5-year survivals in node positive disease compared to node-negative disease were 0.12 (95% CI 0.06 to 0.24) and 0.08 (95% CI 0.03 to 0.22). There was no randomized controlled trial which assessed the survival benefit of routine or “selective” lymphadenectomy. *Conclusion*. Currently, there is no evidence of survival benefit for routine or selective lymphadenectomy. Survival rates are low in patients with positive lymph nodes draining the liver irrespective of whether they are detected by routine lymphadenectomy or by macroscopic involvement. Further trials in this patient group are required.

## 1. BACKGROUND

Colorectal cancer is the third commonest malignancy in the United Kingdom with an estimated 34 000 cases diagnosed every year [1]. In men, it is next only to prostatic and lung cancer and has an incidence of 53.8 per 100 000 population [1]; in women, it is next only to breast and lung cancers and has an incidence of 34 per 100 000 population [1].

It is the second most common cause of cancer mortality (next only to lung cancer) accounting for nearly a tenth of cancer deaths in UK [1] and for 1 in 40 deaths from all causes [1, 2]. Nearly 16 000 people die annually due to colorectal cancer [1].

Liver is the commonest site of recurrence in people who undergo curative surgery for colorectal cancer [3, 4] and 20–32% of these metastatic deposits are respectable [4–6]. The 5-year survival after liver resection varies between 16% and 30% [5, 7–10]. The tumour recurrence rate after resection of hepatic metastases varies between 40.4% and 73.5% [11, 12]. The common sites of recurrence include liver, lung, peritoneum, and locoregional recurrence [12].

Involvement of hepatic lymph nodes during liver resection is considered as a poor prognostic factor [13, 14], with 5-year survival rate after liver resection varying between 0 and 4.3% [8, 15, 16]. In people with positive nodes, after adjusting for different factors such as tumour number [8, 16–18], size [16–18], distribution [16, 17], and surgical resection margin [18], survival rates after liver resection are similar to those in patients with unresectable colorectal metastasis who underwent hepatic infusion chemotherapy [19, 20]. Median survival after systemic chemotherapy with leukovorin, 5-fluorouracil, oxaliplatin, and irinotecan has been recently reported to be around 20 months [21, 22] with an estimated 3-year survival of about 10% [22]. In light of this, hepatic node involvement detected pre-operatively or during surgery is generally considered a contra indication for liver resection for liver secondaries from colorectal primary [23, 24]. Although the surgical technique in the treatment of colorectal liver metastases has undergone few modifications, with the improving results of resection of extra-hepatic disease (5-year survival of 18%) following neo-adjuvant chemotherapy [6], hepatic node involvement as a contra indication for liver resection for colorectal liver metastases requires to be reconsidered.

In patients with liver metastases, about 14% to 15% of nodes (draining the liver) considered uninvolved macroscopically are infiltrated by tumour cells microscopically [15, 16]. Patients who have involvement of common hepatic artery nodes and coeliac artery nodes (considered as group 2 nodes) [25] have been reported to have a poorer prognosis than the patients with involvement of hepato-duodenal or retro-pancreatic group of nodes (considered as group 1 nodes) [25]. Approximately half of the microscopic disease is in the hepato-duodenal and the retro-pancreatic group [25] and therefore amenable to radical lymphadenectomy. The mechanism for development of hepatic node involvement is not known nor whether they represent spread from the liver metastases [8] or the primary bowel cancer.

In the absence of clear beneficial effects of adjuvant chemotherapy in potentially curative liver resections for colorectal liver metastases [26], the practice of adjuvant chemotherapy is varied. While some surgeons use adjuvant chemotherapy after all resections [27, 28], others use adjuvant chemotherapy only in patients with poor prognostic factors [29]. The potential advantage of performing routine hepatic lymphadenectomy at the time of liver resection for colorectal metastases is the removal of the microscopically involved lymph nodes and to provide adjuvant chemotherapy in patients who do not have other poor prognostic factors.

The primary aims of this review are to determine the role of routine lymphadenectomy or lymphadenectomy indicated by macroscopic nodal involvement; to determine the prognostic significance of hepatic lymph node status in patients undergoing liver resection for colorectal metastases; and whether liver resection is indicated in patients with nodal involvement. The secondary aim is to determine if the extent of lymphadenectomy affects prognosis.

A systematic review of the literature on hepatic lymph node involvement in colorectal metastases was published in 2000 [13]. However, in the last 6 years, there have been significant advancements in the treatment of liver metastases including neo-adjuvant chemotherapy [6] and further literature analysis was therefore indicated.

## 2. METHODS

### 2.1. Search strategy

The following databases were searched: Medline (January 1990–January 2006), Embase (January 1990–January 2006), and Central (Issue 1, 2006 from 1990). The following search strategy was used: (“Neoplasm Metastasis” [MeSH] OR metasta* OR secondar* OR spread OR cancer OR carcinoma OR tumour Or tumor OR neoplasm) AND (colon Or colonic OR colorect* OR rectal OR rectum OR gut OR intestine OR bowel OR “Intestine, Large” [MeSH] OR “Colorectal Surgery” [MeSH] OR “Intestinal Neoplasms” [MeSH]) AND ((“Liver” [MeSH] OR “Liver Neoplasms” [MeSH] OR “Liver Diseases” [MeSH] OR liver OR hepatic) AND (segmentectomy OR resection) OR “Hepatectomy” [MeSH]) AND “humans” [MeSH Terms] AND English [Lang] AND (“1990” [PDAT] : “3000” [PDAT]).

Equivalent search strategies were used in the Embase and Central.

### 2.2. Inclusion and exclusion criteria

The following studies were included: randomized controlled trials; comparative series; published in journals January 1990 onwards; full text in English language; should have survival or cancer recurrence data; and a minimum duration of follow up of one year.

The following studies were excluded: number of patients undergoing liver resections <10; includes liver resection for other cancers (primary or secondary); includes repeat or multistaged liver resections; includes combined excision of liver and lung metastases; hepatic lymph-node status not reported; mention of extrahepatic disease but not clearly stated as hepatic lymph nodes; mention of regional lymph nodes but not clear whether the lymph nodes drain the primary tumour or the secondary tumour; not possible to identify the survival or recurrence data for hilar node-positive and negative status separately; no controls (e.g., survival reported only in node-negative disease and node-positive diseases did not undergo resection); lost to follow up >10%.

The following outcomes were measured: in-hospital mortality/surgical mortality/30-day mortality; 1-, 3-, and 5-year survival; 1-, 3-, and 5-year disease-free survival; mean time to recurrence (mean disease free survival).

### 2.3. Data extracted

The following data were extracted from each study using a custom-designed data extraction form: year of publication; year of study; country of study; type of study; population characteristics such as age, gender ratio; inclusion and exclusion criteria used in individual studies; number of individuals entering the study; follow-up period; lost to follow up; tests performed during follow-up period; hepatic lymph node involvement and how it was diagnosed; any adjuvant therapy (including neo-adjuvant therapy)—indication and details; whether routine hepatic lymphadenectomy was performed; operating time; blood loss, number of units transfused; hospital stay; complications of surgery (directly related to surgery—such as bleeding, bile leak, intra-abdominal collections etc., and general complications such as pneumonia; cardiac complications, etc.); survival and mortality data; time to recurrence; type of recurrence (including local or regional or remote in relation to liver and the actual sites of recurrence); and quality of life measures (however, described by author).

### 2.4. Statistical methods

RevMan Analyses 1.0 [30] was used for meta-analysis. The odds ratio with 95% confidence interval was calculated. The random-effects model [31] and the fixed-effect model [32] were used. Subgroup analysis, based on whether the studies included nodes involved macroscopically or microscopically was performed. Other subgroup analyses performed were those based on whether routine lymphadenectomy or selective lymphadenectomy was performed; whether chemotherapy was used, whether only the lymph nodes along the hepatoduodenal ligament were removed (as compared to the removal of the other groups of nodes mentioned previously) and whether a localised or radical lymphadenectomy was performed.

Heterogeneity was explored using the chi-squared test with significance set at P value 0.10 and I^2^ was used to measure the quantity of heterogeneity [33]. StatsDirect 2.4.5 [34] was used for calculating the sample size.

### 2.5. Definitions used

The following definitions were used in this review.

- Disease free survival: patients who are alive and who have not shown any signs of recurrence of cancer clinically or radiologically.
- Primary site: primary site of origin in the colon or rectum.
- Local recurrence: recurrence at the site of liver resection. This has no relation to the recurrence of the tumour at the primary site.
- Regional recurrence: peri-hepatic area including porta hepatis. Again, this has no relation to the regional lymph nodes draining the primary tumour site.
- Remote recurrence: recurrence of cancer in sites not included in the above two categories. In most cases (except in hepatic flexure tumours), recurrence at the primary site will be included in this category.
- Macroscopic lymph node involvement: lymph node involvement as detected radiologically (pre-operatively or per-operatively) or by visual and tactile assessment (Group 3).
- Microscopic lymph node involvement: lymph node involvement not detected radiologically (pre-operatively or per-operatively) or by visual and tactile assessment but detected by microscopic examination (Group 2).
- Lymph node involvement: macroscopic or microscopic lymph node involvement or both (Group 2 or Group 3).
- No lymph node involvement: neither macroscopic nor microscopic lymph node involvement (if assessed) (Group 1).
- Routine lymphadenectomy: lymphadenectomy (of nodes draining the liver) performed routinely in the presence or absence of lymph node involvement.
- Hepatic pedicle nodes: nodes along the hepatoduodenal ligament, retropancreatic, common hepatic artery, and coeliac artery.

## 3. RESULTS

A total of 4230 references through electronic searches of Pubmed (1895), Embase (2222), and Central, The Cochrane Central Register of Controlled Trials in The Cochrane Library (113) were identified. 1403 duplicate references were excluded and 1958 clearly irrelevant references were excluded through reading titles and abstracts. 869 references were retrieved for further assessment. Two more references [35, 36] were identified through scanning reference lists of the retrieved studies. Out of these, 860 studies were excluded because of the exclusion criteria. Eleven reports of ten studies qualified for the review [7, 8, 15–17, 18, 25, 28, 29, 35, 37]. The duplicate report [16, 25] was identified by using the common author and centre. One study, although it met the inclusion criteria, did not provide information for meta-analysis [17]. Thus, nine studies involving 942 patients were included for meta-analysis: 151 (16.0%) were node-positive and the rest were node-negative. There were no randomized controlled trials comparing liver resection alone with liver resection along with routine lymphadenectomy or lymphadenectomy in those with involved nodes. Similarly, there were no randomized controlled trials comparing liver resection with nonsurgical treatments such as chemotherapy in patients with node-positive disease, who were otherwise suitable for liver resection.

The characteristics of included studies and the prevalence of positive nodes in different studies are tabulated in Table 1. The overall prevalence in the included studies was 16.0% and the prevalence was 16.3% in patients who underwent routine lymphadenectomy. The prevalence varied from 5.4% to 50% (see Table 1).

The survival rates of node-positive and node-negative patients for different studies and in different categories are tabulated in Tables 2 and 3. The overall 3-year and 5-year survival rates in node-positive patients were 11.3% and 1.5%, respectively, compared to 3-year and 5-year survival rates of 53.9% and 32.1% in node-negative patients.

**Figure 1:**
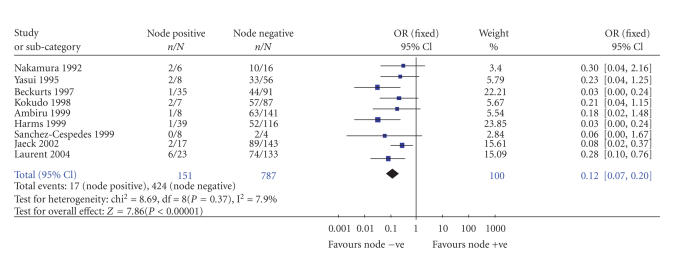


**Figure 2:**
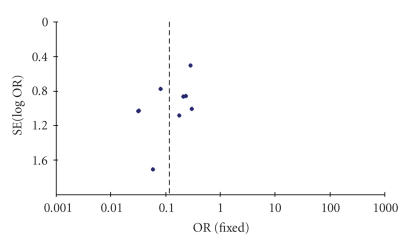


The results of the meta-analysis are tabulated in Table 4. There was significant difference in the odds of one-year (Odds ratio 0.11 95% confidence intervals 0.05 to 0.20), 3-year (OR 0.12 95% CI 0.07 to 0.20), and 5-year survival (OR 0.08 95% CI 0.03 to 0.22) between node-positive and node-negative groups. The results did not change by adopting the fixed or random effects model. The forest plot for 3-year survival is shown in Figure 1. Subgroup analysis was performed for 3-year and 5-year survival. The odds of survival were significantly lower for hepatic node-positive patients compared to node-negative patients in the different subgroup analyses performed, for example, including studies published before 2000; studies published after 2000; including only studies with microscopic nodal involvement; only studies in which chemotherapy was used; and different groups classified by the extent of dissection. Visual assessment of the funnel plot did not demonstrate any bias (see Figure 2).

Only one study [18] reported the disease-free survival rate. There was no statistically significant difference in the proportion of node-positive and node-negative patients who were disease free at one, three, and five years after liver resection. None of the studies reported on quality of life measures.

There was no significant heterogeneity for the important outcomes studied as indicated by the chi-square test, I^2^ value, and the similar odd's ratio using the fixed and random effects model. The only outcomes with significant heterogeneity were the one-year survival and the 3-year survival in the subgroup analysis for routine lymphadenectomy and chemotherapy.

## 4. DISCUSSION

The overall prevalence of positive lymph nodes (draining the liver) was 16.0%. However, the prevalence varied between 5.4% and 50%. The difference in the prevalence could reflect the extent of lymphadenectomy. Some surgeons remove only the nodes along the hepatoduodenal ligament [7, 8, 29], whilst others remove nodes along the common hepatic artery, coeliac artery, and the retropancreatic area [16]. In the study which reported the more extensive lymphadenectomy, the prevalence of the nodes along the hepatoduodenal ligament and retropancreatic area was 5% and an additional 5.6% of patients had involvement of the nodes along the common hepatic artery and coeliac artery. However, two studies [8, 29] which performed a less-extensive lymphadenectomy (hepatoduodenal ligament group only) found a prevalence of greater than 25%. Thus, the extent of the lymphadenectomy is not the main factor that causes the difference in the prevalence rate between studies. Factors that may influence the prevalence are patient selection for surgery, the adequacy of pre-operative staging; whether nodal macroscopic disease was excluded from the study, number of nodes examined [37], number of sections per node and use of special techniques to identify microscopic involvement [37].

There was significant heterogeneity between the studies in the one year survival with a 50% difference between the study with maximum and that with minimum survival. This is more likely to reflect different criteria used for selecting patients for “potentially curative resection” rather than surgical techniques. In spite of the difference in the prevalence of node-positive cases and the selection criteria, the data was suitable for meta-analysis because of the lack of heterogeneity in most of the 3-year and 5-year survival comparisons.

No significant difference was found in the proportion of hepatic node-positive and node-negative patients who were disease free at the end of one, three, and five years following lymphadenectomy. This is surprising in view of the major difference in survival and may reflect the small number of patients in the node-positive category. Only one study [18] reported this outcome.

The meta-analysis has clearly demonstrated that the survival rates are lower in patients with positive hepatic lymph nodes compared to patients with negative lymph nodes, irrespective of whether the involvement was microscopic or macroscopic and whether routine or selective lymphadenectomy was carried out. The odds of 3-year survival in patients with positive lymph nodes (draining the liver) were about an eighth of the odds of survival in patients with negative lymph nodes. This dropped even more to one twelfth for 5-year survival in patients with positive lymph nodes compared to those with negative lymph nodes. Only 2 of the 151 patients with positive hepatic node were alive at 5 years.

Two studies [15, 35] compared survival in microscopic involvement of hepatic pedicle (hepatoduodenal and common hepatic artery group of nodes) having excluded those who had macroscopic involvement). The survival was significantly lower in node-positive group compared to the node-negative group. The 3-year and 5-year survival of microscopic node-positive patients were 20.5% and 3.2% compared to 56.5% and 26.5% in microscopically node-negative disease. Since the survival data on patients with macroscopic disease is not available separately, it is not clear whether there is a survival difference between macroscopic and microscopic hepatic lymph node involvement.

It is not clear from many studies whether the nodes with macroscopic involvement were confirmed histologically. The study by Beckhurts et al. demonstrated that one third of the nodes that appear to be macroscopically involved on imaging (3 out of 9 patients with CT evidence of hepatic nodal involvement) were found to be histologically negative for metastatic spread [8]. Clearly, patients require histological confirmation of suspected nodal disease.

The 3-year survival and 5-year survival in positive hepatoduodenal ligament nodal status was 4.7% and 1.2% compared to 3-year and 5-year survival rates of 11.3% and 1.5% in patients with positive hepatic pedicle nodal status. The latter group by definition includes the former group also. However, in the studies where the hepatic pedicle was dissected, the survival data for patients with common hepatic artery and coeliac node involvement is not available separately from that of patients with hepatoduodenal ligament nodal involvement. Whether involvement of common hepatic artery and coeliac artery nodes carries a worse prognosis compared to hepatoduodenal ligament nodal involvement has not been established nor whether a more extensive lymphadenectomy confers benefit.

There have been no randomized controlled trials comparing liver resection alone with routine lymphadenectomy or lymphadenectomy in those with macroscopically involved nodes. Similarly, there have been no randomized controlled trials comparing liver resection along with lymphadenectomy with nonsurgical treatments such as chemotherapy in patients with node-positive disease, who were otherwise suitable for liver resection.Routine lymphadenectomy is being performed to remove the microscopically involved nodes draining the liver and provide cancer clearance. Although none of the included studies reported any complication specifically related to routine lymphadenectomy, it involves removing additional structures. Radical lymphadenectomy in patients with other gastrointestinal cancers has been reported to be associated with increased morbidity [38]. None of the included studies compared the survival or recurrence rates between those who underwent routine lymphadenectomy and selective lymphadenectomy (based on finding macroscopic disease). However, the 5-year survival of node-positive patients identified by routine lymphadenectomy is only 2/130, for example, 1.5%. Node-negative patients from the same cohort of patients who underwent potentially curative liver resection had a 5-year survival of 29.9%. This suggests that node-positive disease had a major impact on cancer recurrence.

Two of the included studies, involving patients who underwent routine lymphadenectomy, used adjuvant chemotherapy. The 3-year and 5-year survival in hepatic node-positive patients in these two studies were 6.7% and 0%. In the study by Nakamura et al. [28], all the patients in the study received chemotherapy. The 3-year survival in the 6 patients with positive hepatic lymph node was 33.3%. This study did not report the 5-year survival. The second study which mentioned the chemotherapy was by Harms et al. [29], in which chemotherapy was given in patients with poor prognostic factors. The 3-year and 5-year survival of hepatic node-positive cases in this study was 2.6% and 0%.There is currently no evidence of any benefit of adjuvant chemotherapy in the presence of nodal disease.

None of the studies considered the role for neo-adjuvant chemotherapy. Recently, neo-adjuvant chemotherapy has been used to downstage previously non-resectable metastatic colorectal cancer (the reasons for nonresectability include extra-hepatic disease in the lungs and lymph nodes) with reasonable three-year results [5, 39]. Preoperative chemotherapy is being used in other node-positive tumours including oesophageal cancers [40, 41] and rectal cancers [42, 43] for downstaging the disease and may improve the median survival [40]. It is not clear whether neo-adjuvant chemotherapy will be useful in improving the survival in people with node-positive disease. The role of neo-adjuvant chemotherapy in patients with colorectal liver metastases with hepatic node involvement needs to be evaluated in a prospective trial. However, only people with macroscopic positive hepatic lymph nodes could be included in this trial. The incidence of macroscopically positive hepatic lymph nodes is 4.8% in patients with otherwise resectable colorectal hepatic metastases [8]. In order to demonstrate a 5% 3-year survival benefit (an additional 5% of people surviving at the end of 3 years with the control 3-year survival rate assumed to be 11.3%; see Table 3), the overall sample size required is 36 patients when the alpha and beta errors were set at 0.05 and 0.2. However, in such a study, there will a high percentage of cross-over between the groups as patients, who progress in spite of chemotherapy in the surgery group may no longer be resectable. It may also be unethical to refuse surgery for patients belonging to the chemotherapy group, whose disease has been down staged by chemotherapy. However, these patients could be considered for a study involving surgical resection for patients responding to systemic chemotherapy.

A better 3-year survival in patients with nodal disease was noted in the studies published after 2000 (8/40 = 20%) compared to those published before 2000 (9/111 = 8.1%). However, the 5-year survival in patients with nodal disease is poor in both categories. It is possible that the improved adjuvant chemotherapy has an impact on survival in patients with nodal disease.

From the results of our review, we conclude that there is no evidence of survival benefit for routine lymphadenectomy from randomized controlled trials or from observational studies comparing routine and selective lymphadenectomy at the time of liver resection for colorectal liver metastases. Patients with enlarged hilar lymph nodes require histological confirmation of metastatic disease. Patients with confirmed hepatic node-positive status have a poor prognosis in spite of resection with few 3-year survivors (11.3%). Survival rates are low in patients with positive lymph nodes draining the liver irrespective of whether they are detected by routine lymphadenectomy or by macroscopic involvement. Patients with resectable liver metastases and combined hilar nodal involvement could be considered for a study offering surgical resection (liver resection and lymphadenectomy) for patients responding to systemic chemotherapy.

## Figures and Tables

**Table 1:** Study characteristics.

Study name	Study characteristics (country of study, year of study)	Number of people included for the review	Number of node-positive cases *	Population characteristics (age in years, -mean, range, gender ratio M/F)	Routine or Selective lympha-denectomy	Microscopic or macroscopic involvement or both	Group of nodes dissected	Chemotherapy	Indications for chemotherapy
Nakamura et al. [28]	Japan 1978–1990	22	6 (27.3%)	Not available	Routine	Microscopic	Hepatic pedicle (hepatoduodenal ligament, retropancreatic and celiac axis)	Adjuvant systemic chemotherapy	All

Yasui et al. [35]	Japan 1983–1994	64	8 (12.5%)	Not available	Routine	Both	Hepatic pedicle (hepatoduodenal ligament, retropancreatic, hepatic artery)	Not stated	Not stated

Beckhurts et al. [8]	Germany 1987–1994	126	35 (27.8%)	Not available	Routine	Both	Hepatoduodenal ligament	Not stated	Not stated

Kokudo et al. [18]	Japan 1980–1997	94	7 (7.4%)	Not available	Not stated	—	Hepatic hilum	Adjuvant local or systemic chemotherapy or no chemotherapy	Not stated

Ambiru et al. [7]	Japan 1984–1997	149	8 (5.4%)	Not available	Routine	Both	Hepatoduodenal ligament	Adjuvant local chemotherapy or no chemotherapy	Not stated

Harms et al. [29]	Germany 1987–1998	155	39 (25.2%)	Not available	Routine	Both	Hepatoduodenal ligament	Adjuvant local or systemic chemotherapy or no chemotherapy	Poor prognostic factors

Sanchez-Cespedes et al. [37]	USA	16^†^	8 (50%)	Not available	Not stated	Microscopic (molecular analysis)	Perihepatic nodes	Not stated	Not stated

Jaeck et al. [16, 25]	France 1993–98	160	17 (10.6%)	94 Males/66 Females	Routine	Both	Hepatic pedicle (Hepatoduodenal, retropancreatic, celiac axis, hepatic artery)	Not stated	Not stated

Laurent et al. [15]	France 1985–2000	156	23 (14.7%)	106 males/50 females 64 years (median) 24–86 years (range)	Routine	Microscopic	Hepatic pedicle (hepatoduodenal, hepatic artery)	Adjuvant systemic chemotherapy	Not stated

Total		942	151 (16.0%)						

* Percentages in brackets;

^†^ No follow-up data available for one patient

**Table 2:** Survival in each study*.

Study	Node-positive				Node-negative			
	Total	One-year survival	Three-year survival	Five-year survival	Total	One-year survival	Three-year survival	Five-year survival
Nakamura et al. [28]	6	—	2 (33.3%)	—	16	—	10 (62.5%)	—
Yasui et al. [35]	8	—	2 (25%)	0 (0%)	56	—	33 (58.9%)	18 (32.1%)
Beckhurts et al. [8]	35	—	1 (2.9%)	0 (0%)	91	—	44 (48.4%)	20 (22.0%)
Kokudo et al. [18]	7	6 (85.7%)	2 (28.6%)	0 (0%)	87	84 (96.6%)	57 (65.5%)	47 (54.0%)
Ambiru et al. [7]	8	—	1 (12.5%)	1 (12.5%)	141	—	63 (44.7%)	38 (27.0%)
Harms et al. [29]	39	—	1 (2.6%)	0 (0%)	116	—	52 (44.8%)	24 (20.7%)
Sanchez-Cespedes et al. [37]	8	2 (25%)	0 (0%)	—	7	7	2/4^†^ (50%)	—
Jaeck et al. [16, 25]	17	4 (23.5%)	2 (11.8%)	0 (0%)	143	135 (94.4%)	89 (62.2%)	67 (46.9%)
Laurent et al. [15]	23	16 (69.6%)	6 (26.1%)	1 (4.3%)	133	117 (88%)	74 (55.6%)	32 (24.1%)

Total	151	28/55 (50.9%)	17/151 (11.3%)	2/137 (1.5%)	790	343/370 (92.7%)	424/787 (53.9%)	246/767 (32.1%)

* Percentages in brackets;

^†^ The follow-up for the other 3 patients was <3 years

**Table 3:** Survival in each category.

Category	Number of studies	Node-positive		Node-negative	
		Total number	Number survived	Total number	Number survived
**Three-year survival**					

All studies	9	151	17 (11.3%)	787	424 (53.9%)

Studies including microscopic hepatic node involvement	3	39	8 (20.5%)	193	109 (56.5%)

Studies where routine lymphadenectomy was performed irrespective of the macroscopic nodal status	7	136	15 (11.0%)	696	365 (52.4%)

Studies which involved clearance of perihepatic nodes or hilar nodes only	2	15	2 (13.3%)	91	59 (64.8%)

Studies which involved clearance of nodes along the hepatoduodenal ligament only (as opposed to clearance of the retropancreatic group, common hepatic artery group, and celiac group)	3	82	3 (4.7%)	348	160 (46.0%)

Studies which involved clearance of all the groups of nodes in the hepatic pedicle	4	151	17 (11.3%)	787	424 (53.9%)

Studies in which chemotherapy was used	2	45	3 (6.7%)	132	62 (47.0%)

Studies published before 2000	7	111	9 (8.1%)	511	261 (51.1%)

Studies published after 2000	2	40	8 (20%)	276	163 (59.1%)

**Five-year Survival**					

All studies	7	137	2 (1.5%)	767	246 (32.1%)

Studies including microscopic hepatic node involvement	2	31	1 (3.2%)	189	50 (26.5%)

Studies where routine lymphadenectomy was performed irrespective of the macroscopic nodal status	6	130	2 (1.5%)	680	199 (29.3%)

Studies which involved clearance of perihepatic nodes or hilar nodes only	1	7	0 (0%)	47	87 (54.0%)

Studies which involved clearance of nodes along the hepatoduodenal ligament only (as opposed to clearance of the retropancreatic group, common hepatic artery, group and celiac group)	3	82	1 (1.2%)	348	82 (23.6%)

Studies which involved clearance of all the groups of nodes in the hepatic pedicle	3	137	2 (1.5%)	767	246 (32.1%)

Studies in which chemotherapy was used	1	39	0 (0%)	116	24 (20.7%)

Studies published before 2000	5	97	1 (1.0%)	491	147 (29.9%)

Studies published after 2000	2	40	1 (2.5%)	276	99 (35.9%)

**Table 4:** Results of meta-analysis.

Studies included	Number of studies included	Participants	Odds ratio [95% confidence intervals]	Statistical significance	Heterogeneity (Higgins' I^2^)
**One-year survival**					

All	4	425	0.08 [0.01, 0.47]^†^	Significant	75.1%

**Three-year survival**					

All	9	938	0.15 [0.08, 0.26]	Significant	7.9%
Microscopic node involvement	3	232	0.24 [0.11, 0.55]	Significant	0%
Routine lymphadenectomy	7	832	0.13 [0.06, 0.28]5^†^	Significant	27.3%
Hepatoduodenal ligament only	3	430	0.24 [0.11, 0.55]	Significant	0%
Hepatic pedicle	4	402	0.20 [0.10, 0.39]	Significant	0%
Adjuvant chemotherapy	2	177	0.10 [0.01, 1.03]^†^	Not significant	63.5%
Published before 2000	7	622	0.09 [0.04, 0.18]	Significant	1.8%
Published after 2000	2	316	0.17 [0.05, 0.57]^†^	Significant	46.1%

**Five-year survival**					

All	7	904	0.10 [0.04, 0.27]	Significant	0%
Microscopic node involvement	2	220	0.14 [0.03, 0.72]	Significant	0%
Routine lymphadenectomy	6	810	0.09 [0.03, 0.24]	Significant	0%
Hepatoduodenal ligament only	3	430	0.09 [0.02, 0.38]	Significant	0%
Hepatic pedicle	3	380	0.08 [0.02, 0.34]	Significant	0%
Adjuvant chemotherapy	1	155	0.05 [0.00, 0.81]	Significant	Not applicable
Published before 2000	5	588	0.09 [0.03, 0.29]	Significant	0%
Published after 2000	2	316	0.07 [0.01, 0.38]	Significant	0%

**One-year disease-free survival**					

All	1	94	0.24 [0.05, 1.15]	Not significant	Not applicable

**Three-year disease-free survival**					

All	1	94	0.10 [0.01, 1.78]	Not significant	Not applicable

**Five-year disease-free survival**					

All	1	94	0.15 [0.01, 2.81]	Not significant	Not applicable

^†^ Random-effects model as there was statistical heterogeneity.
